# SAM, SAH and *C. elegans* longevity: insights from a partial AHCY deficiency model

**DOI:** 10.1038/s41514-023-00125-1

**Published:** 2023-12-05

**Authors:** Pankaj Thapa, Katarzyna Olek, Agata Kowalska, Remigiusz A. Serwa, Wojciech Pokrzywa

**Affiliations:** 1 Laboratory of Protein Metabolism, International Institute of Molecular and Cell Biology in Warsaw, Warsaw, Poland; 2 IMol Polish Academy of Sciences, Warsaw, Poland

**Keywords:** Ageing, Metabolic syndrome

## Abstract

Supplementation with S-adenosylhomocysteine (SAH) extends the lifespan of model organisms. To explore the impact of SAH on aging, we generated a *Caenorhabditis elegans* model by introducing the S-adenosylhomocysteine hydrolase (AHCY-1) variant Y145C, corresponding to the human AHCY Y143C pathogenic mutation. This mutation is anticipated to impair SAH hydrolysis, resulting in its increased levels. Our findings revealed that animals with this endogenous mutation exhibited delayed aging, accompanied by decreased S-adenosylmethionine (SAM) and moderately increased SAH levels. The extended lifespan of these worms depends on the AMP-activated protein kinase (AMPK), its activator Vaccinia virus–related kinase (VRK-1), and the DAF-16 transcription factor. The results underline the complex nature of SAH’s influence on aging, proposing that the balance between SAM and SAH might play a pivotal role in defining the lifespan of *C. elegans*. Moreover, our partial AHCY-1 deficiency model offers a tool for studying the intersection of methionine metabolism and aging.

## Introduction

**Fig. 1:**
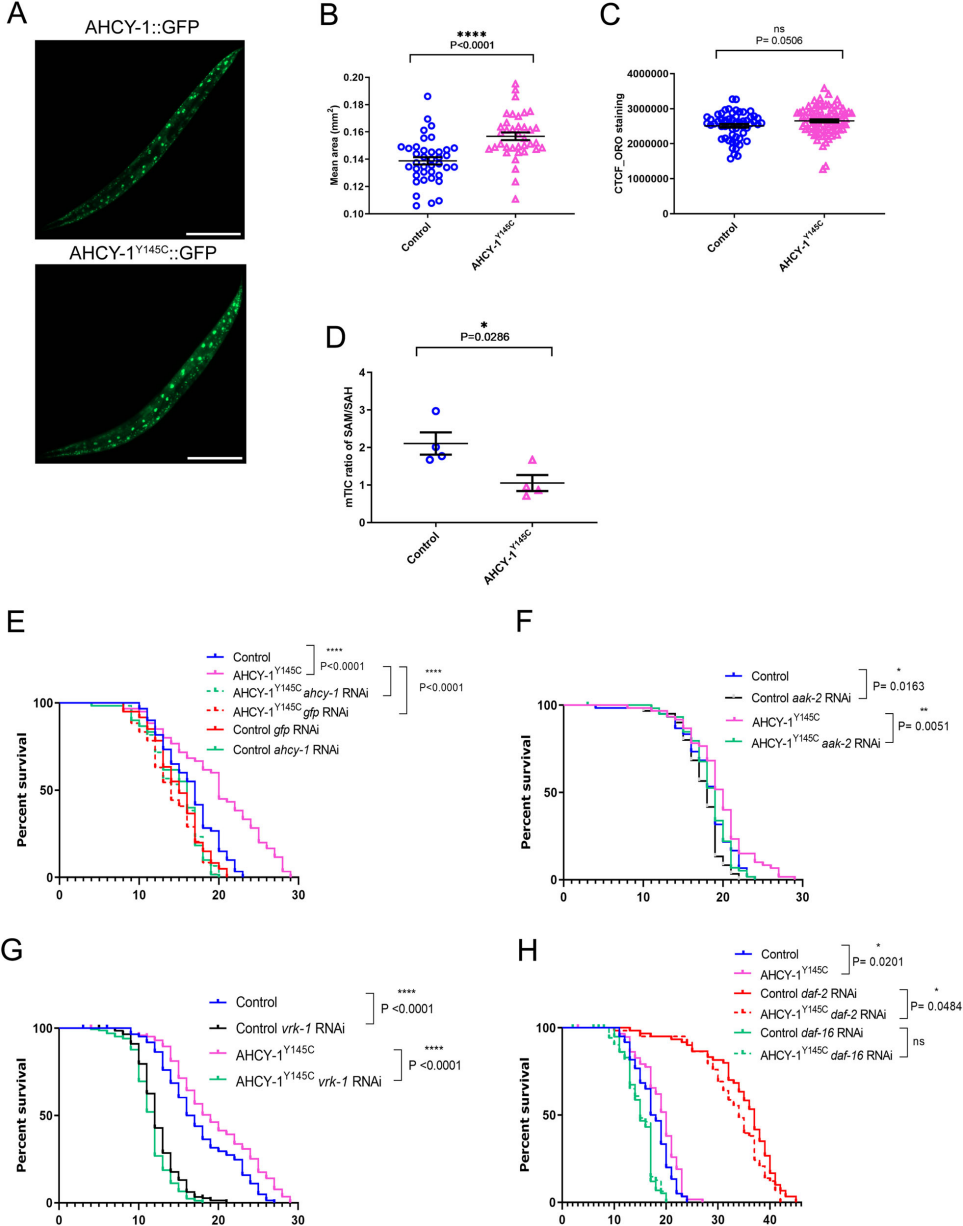
Exploring the AHCY-1^Y145C^ mutation and its effects in *C. elegans*. **A** GFP tagged AHCY-1 and AHCY-1^Y145C^ visualized through fluorescence microscopy. The scale bar represents 200 µm. **B** Comparison of the mean area between control (AHCY-1::GFP) and AHCY-1^Y145C^ animals. Analysis based on 37–39 worms from three biological repeats. Statistical significance determined using the Mann–Whitney test. **C** Lipid content quantification in young adult control and AHCY-1^Y145C^ worms using the ORO staining method. Higher corrected total cell fluorescence (CTCF) signifies greater lipid content. Analysis based on 24–42 worms from three biological repeats. Statistical significance determined using the Mann–Whitney test. **D** The liquid chromatography with tandem mass spectrometry (LC-MS/MS) method was used to measure the levels of SAM, SAH (represented as mTIC-normalized peak intensities), and the ratio of SAM to SAH in control (AHCY-1::GFP) and AHCY-1^Y145C^ mutant worm lysates. The mean and SEM were calculated from four biological repeats. **E** Lifespan comparisons of control and AHCY-1^Y145C^ worms subjected to *ahcy-1* or *gfp* RNAi at 20 °C. Statistical significance was determined using the Mantel-Cox log-rank test. **F** Lifespan comparisons of control and AHCY-1^Y145C^ worms subjected to *aak-2* RNAi at 20 °C. Statistical significance determined using the Mantel-Cox log-rank test. **G** Lifespan comparisons of control and AHCY-1^Y145C^ worms subjected to *vrk-1* RNAi at 20 °C. Statistical significance was determined using the Mantel-Cox log-rank test. **H** Lifespan comparisons of control and AHCY-1^Y145C^ worms subjected to *daf-2* and *daf-16* RNAi at 20 °C. Statistical significance was determined using the Mantel-Cox log-rank test.

S-adenosylhomocysteine (SAH) is a key intermediate in the methionine cycle formed by S-adenosylmethionine (SAM) demethylation. Following its formation, SAH is converted into homocysteine and adenosine by the conserved enzyme S-adenosylhomocysteine hydrolase (AHCY)^1^. Pathogenic variants of AHCY that affect its activity and result in increased cellular levels of SAH are associated with a rare autosomal recessive disorder of methionine metabolism^2,3^. Interestingly, SAH treatment increased lifespan in yeast and worms through an AMP-activated protein kinase (AMPK)-dependent mechanism, partially by inducing a methionine restriction state, known to improve healthspan in most model organisms^4–9^. By exploiting the equivalent pathogenic mutation in the gene encoding *Caenorhabditis elegans* AHCY (AHCY-1), we aimed to demonstrate the link between a decline in AHCY-1 activity and the resulting increase in intracellular SAH and aging. Specifically, we generated animals producing AHCY-1 variants Y145C and Y320D corresponding to human AHCY Y143C and Y328D pathogenic mutations, respectively (Supplementary Fig. 1A), using the CRISPR/Cas9 system. AHCY-1 depletion and the Y320D mutation proved lethal for worms, in contrast to the Y145C, which we subsequently tagged with GFP (CRISPR/Cas9 knock-in) to monitor its localization (hereafter AHCY-1^Y145C^). As we showed earlier, the GFP tag does not affect the functionality of wild-type AHCY-1^10^. AHCY-1^Y145C^ displayed similar localization to wild-type AHCY-1, with a potent signal in cell nuclei in the worm body (Fig. 1A). We then examined the phenotypes of AHCY-1^Y145C^- producing animals. Using the WormLab system (MBF Bioscience), we noted that the body size of AHCY-1 mutants is larger than that of control animals, suggesting that AHCY-1 is involved in the regulation of worm development (Fig. 1B). Comprehensive assessments of the animals’ locomotor characteristics did not indicate impairment in AHCY-1^Y145C^ mobility (Supplementary Fig. 1B). AHCY-1^Y145C^ animals were also fertile and showed a similar number of laid/hatched eggs to control animals (Supplementary Fig. 1C). In parallel, via quantitative measurements of the proteome, we found no significant changes in the proteome of AHCY-1^Y145C^ worms compared to the control (Supplementary Fig. 1D and Supplementary Table 1). Previously, we showed that AHCY-1 depletion by RNA interference (RNAi) increases lipid abundance in worms^10^. While the total lipid content did not show a significant difference between control and AHCY-1^Y145C^ worms as per Oil Red O (ORO) staining, a *P*-value equal to 0.0506 hints at a potential subtle disruption in AHCY-1^Y145C^ function (Fig. 1C). To assess the impact of this mutation on the partial impairment of AHCY-1, we undertook metabolomic analysis to gauge the levels of SAM and SAH in young adult worms. As anticipated, we found a decrease in SAM and a slight increase in SAH levels in AHCY-1^Y145C^ worms, leading to an increased SAH to SAM ratio in these mutants (Fig. 1D and Supplementary Fig. 1E). To evaluate the consequences of these changes, we measured the longevity of these mutants compared to the control worms. We observed that the lifespan of worms expressing the AHCY-1^Y145C^ variant was indeed prolonged. This increase was AHCY-1-dependent, as evidenced by the decrease in survival when AHCY-1 was depleted (*ahcy-1* or *gfp* RNAi) (Fig. 1E and Supplementary Table 2). Our immunoblot analyses demonstrate that RNAi treatments result in only a modest reduction in AHCY-1 levels (Supplementary Fig. 1F). While this decrement appears insufficient to affect the lifespan of control worms, it markedly diminishes the lifespan of Y145C mutants. This observation likely underscores the different thresholds of AHCY-1 activity present in these unique genetic contexts. RNAi knockdown of AAK-2/AMPK demonstrated a considerable impact on the lifespan of AHCY-1^Y145C^ worms, paralleling the observations seen in SAH-treated animals^7,8^. Further experiments underscored that the mutant’s longevity is intricately tied to the AMPK pathway and its upstream activator, Vaccinia virus–related kinase (VRK-1)^11^ (Fig. 1F, G and Supplementary Table 2). We also noticed that the lifespan of AHCY-1^Y145C^ worms is shortened following DAF-16 knockdown, and the impact of DAF-2 RNAi on lifespan extension is less significant in these mutants, underscoring the pivotal role of insulin signaling (IS) in the longevity mediated by AHCY-1^Y145C^ (Fig. 1H and Supplementary Table 2).

In conclusion, we have developed a *C. elegans* model with partial AHCY-1 deficiency (Y145C mutation), providing a unique lens to explore AHCY’s roles. Y145C mutation sustains enough AHCY-1 activity for survival, yet impacts the worm’s metabolic landscape and aging rate. When we subjected AHCY-1^Y145C^ mutants to *ahcy-1* RNAi, this probably further diminished AHCY-1 activity below a critical threshold, triggering compensatory mechanisms or additional metabolic shifts that ultimately influence lifespan. Our data indicate that RNAi treatments only modestly decrease AHCY-1 levels, which may not be sufficient to impact the lifespan of control worms notably. This aligns with previous findings in *Drosophila melanogaster*^12^, where significant reduction of dAhcy level via whole-body or neuron-specific RNAi dramatically increases SAH levels and, in turn, reduces longevity. While individual SAH levels in AHCY-1^Y145C^ mutant *C. elegans* might not mirror the dramatic shifts observed with full knockdowns in other systems, the significant alteration in the SAM/SAH ratio stands out. This marked shift, especially when factoring in the presence of a protein with a pathogenic point mutation, underscores the multifaceted nature of methionine metabolism. This correlation between SAH accumulation and longevity underscores the delicate balance of AHCY-1 activity needed for normal aging. Common longevity-enhancing strategies, such as downregulating IS via *daf-2* RNAi, appear less beneficial in AHCY-1^Y145C^ worms. This could be due to potential disruptions in methionine metabolism in the context of diminished IS, as suggested by Tain et al.^13^. The altered SAH metabolism in AHCY-1^Y145C^ worms might further complicate this metabolic pathway. Additionally, our findings suggest that while AMPK, especially AAK-2, plays a role in AHCY-1^Y^^145C^ longevity, the kinase VRK-1 also stands out as a significant contributor, potentially through the AMPK activation. This data supports the interconnectedness of SAH breakdown, IS, and AMPK pathways in managing longevity. Our research emphasizes the importance of maintaining equilibrium in AHCY-1 activity for aging processes, proposing that a delicate balance in SAM and SAH levels could be a decisive factor in determining the lifespan of *C. elegans*.

## Methods

### *C. elegans* strains

Worms were maintained on nematode growth medium (NGM) plates seeded with OP50 *Escherichia coli* bacteria at 20 °C unless otherwise stated. PHX646 (ahcy‐1(syb646[ahcy‐1::GFP]I) strain was used as control^10^. The strain generated in this study, PHX784 (syb784 syb646 [ahcy‐1Y145C::GFP]I), was produced by SunyBiotech using CRISPR services (http://www.sunybiotech.com). PHX646 and PHX784 were outcrossed 2× to N2 to generate WOP122 and WOP159 strains, respectively.

### Worm mobility and size

Approximately 10 young adult worms per replicate were placed onto NGM plates and were recorded for 2 min using the WormLab system (MBF Bioscience). The frame rate, exposure time, and gain were set to 7.5 frames per second, 0.0031 s, and 1, respectively. The track length and size of the individual worm were analyzed using the WormLab software (MBF Bioscience). The assay consisted of three independent biological replicates. 37–39 worms were recorded for one biological replicate.

### RNA interference

RNA interference in *C*. *elegans* was performed using the standard RNAi feeding method^14^. For experiments, NGM plates supplemented with 1 mM IPTG and 25 µg/µl carbenicillin seeded with HT115 *E. coli* bacteria expressing double-stranded RNA (dsRNA) against the gene of interest or, as a control, bacteria with the empty vector were used. Worms were placed on freshly prepared RNAi plates as L4 larvae.

### Lifespan assay

All lifespan measurements were done from the L4 stage at 20 °C on NGM plates containing 400 µM FUdR. During lifespan measurements, worms were scored daily for movement and pharyngeal pumping until death. Animals that crawled off the plate or exhibited baggy phenotype were censored from the experiment. Data was analyzed using GraphPad Prism 9 software and the Log-Rank (Mantel-Cox) test. Statistical data of individual lifespan experiments is presented in Supplementary Table 2. The experiments were not randomized. No statistical methods were used to predetermine the sample size. The investigators were blinded to allocation during experiments.

### Egg-laying and hatching analysis

To conduct a detailed analysis of the egg-laying and hatching patterns of wild-type and AHCY-1^Y145C^ mutant *C*. *elegans*, we maintained the strains under standardized conditions until they reached adulthood. Beginning on the first day of adulthood, three worms from each strain were isolated and placed individually on fresh plates; this procedure was repeated every 24 h to observe their reproductive behaviors throughout their reproductive phase. Each day, we counted the number of eggs laid on the respective previous plates to acquire a precise daily egg-laying frequency. In tandem, we noted the number of hatched larvae to gauge the hatching success rate daily. Following this, we aggregated the daily egg counts to estimate the cumulative egg output during the reproductive period, creating a data-rich portrait of both strains’ reproductive patterns. This data was then analyzed using GraphPad Prism 9 software, with statistical comparisons facilitated through Sidak’s multiple comparisons test.

### Protein isolation and immunoblotting

Worms were harvested, washed, and centrifuged at 800 g with multiple wash cycles. Pelleted worms were resuspended and subjected to a freeze-thaw method in liquid nitrogen to rupture the cuticle and liberate intracellular proteins. Afterward, worm pellets were incubated at 95 °C with a sample buffer, sonicated on ice, and centrifuged to remove debris. Extracted protein supernatants were immediately used for protein quantification or stored at −80 °C. Protein concentrations were determined using the Pierce BCA Protein Assay Kit and balanced for uniform protein quantities across samples. These samples were loaded into 10% polyacrylamide gel wells alongside the PageRuler Plus Prestained Protein Ladder. The gel was run at varying voltages, followed by a wet transfer of proteins to a PVDF membrane. Post transfer, blots were blocked and then incubated with primary antibodies, namely Monoclonal anti-α-tubulin antibody (Sigma-Aldrich, T6074, 1:10 000 dilution) and Anti-Green Fluorescent Protein (Bio-Rad, AHP975, 1:2000 dilution), overnight at 4 °C. Following multiple washes, blots were treated with diluted secondary antibodies for 90 min. After a final washing step, the blots were incubated in a homemade ECL solution for chemiluminescent detection. The relative quantification of AHCY-1 bands against a standard protein amount was conducted using Bio-Rad ImageLab software. All blots were derived from the same experiment and processed in parallel.

### Metabolomics

#### Chemicals

LC-MS grade water, acetonitrile, and methanol were obtained from Th. Geyer (Germany). High-purity ammonium formate and formic acid were purchased from Merck (Germany). For internal standards, a labeled amino acid mixture (MSK-A2-1.2; Cambridge Isotope Laboratories, MA, USA) was used at a final concentration of 2%.

### Sample preparation

After centrifugation, 7000 young adult worms were harvested and washed three times with M9 buffer to remove residual bacterial food and snap frozen in liquid N2. Snap-frozen pellet was further centrifuged for 10 min at 15,000 *g* and 4 °C with a 5415 R microcentrifuge (Eppendorf, Hamburg, Germany); a residual buffer was removed without disturbing the pellet, and samples were dried under a stream of nitrogen. After the addition of 300 µL 80% methanol, samples were homogenized on dry ice via a bead beater (FastPrep-24; MP Biomedicals, CA, USA) at 6.0 m/s (3 × 30 s, 5 min pause time) using 1.0 mm zirconia/glass beads (Biospec Products, OK, USA). The homogenized metabolite extracts were centrifuged, supernatants were transferred to analytical glass vials, and the LC-MS/MS analysis was initiated within one hour after the completion of the sample preparation.

### LC-MS/MS analysis

LC-MS/MS analysis was performed on a Vanquish UHPLC system coupled to Orbitrap Exploris 240 high-resolution mass spectrometer (Thermo Scientific, MA, USA) in positive ESI (electrospray ionization) mode. Chromatographic separation was performed on an Atlantis Premier BEH Z-HILIC column (Waters, MA, USA; 2.1 mm × 100 mm, 1.7 µm) at a 0.25 mL/min flow rate. The mobile phase consisted of water: acetonitrile (9:1, v/v; mobile phase A) and acetonitrile: water (9:1, v/v; mobile phase B), modified with a total buffer concentration of 10 mM ammonium formate. The aqueous portion of each mobile phase was adjusted to pH 3.0 with the addition of formic acid. The following gradient (20 min total run time including re-equilibration) was applied (time [min]/%B): 0/95, 2/95, 14.5/60, 16/60, 16.5/95, 20/95. The column temperature was maintained at 40 °C. The autosampler was set to 4 °C, and the sample injection volume was 4 µL. Analytes were recorded via a full scan with a mass resolving power of 120,000 over a mass range from 60 to 900 *m/z* (scan time: 100 ms, RF lens: 70%). The data-dependant acquisition was carried out to obtain MS/MS fragment spectra (resolving power: 15,000; scan time: 22 ms; stepped collision energies [%]: 30/50/70; cycle time: 900 ms). Ion source parameters were set to the following values: spray voltage: 3500 V, sheath gas: 30 psi, auxiliary gas: 5 psi, sweep gas: 0 psi, ion transfer tube temperature: 350 °C, vaporizer temperature: 300 °C.

All experimental samples were measured in a randomized manner. Pooled quality control (QC) samples were prepared by mixing equal aliquots from each processed sample. Multiple QCs were injected at the beginning of the analysis in order to equilibrate the analytical system. QC sample was analyzed after every 5^th^ experimental sample to monitor instrument performance throughout the sequence. An additional processed blank sample was recorded to determine background signals and subsequent background subtraction. Data were processed using MS‑DIAL, and raw peak intensity data were normalized via the total ion count of all detected analytes. Feature identification was based on accurate mass, isotope pattern, MS/MS fragment scoring, and retention time matching to an in‑house library (level 1 confidence identification).

### Proteomics

*C. elegans* were extracted using the Sample Preparation by Easy Extraction and Digestion (SPEED) protocol^15^. In brief, *C. elegans* were solubilized in concentrated TFA (cell pellet/TFA 1:2-1:4 (v/v)) and incubated for 2–10 minutes at room temperature. Samples were neutralized with 2 M Tris-Base buffer using 10 × volume of TFA and further incubated at 95 °C for 5 min after adding Tris(2-carboxyethyl)phosphine (final concentration 10 mM) and 2-chloroacetamide (final concentration 40 mM). Turbidity measurements determined protein concentrations at 360 nm, adjusted to the same concentration using a sample dilution buffer (2 M TrisBase/TFA 10:1 (v/v)), and then diluted 1:4–1:5 with water. Digestion was carried out overnight at 37 °C using trypsin at a protein/enzyme ratio of 100:1. TFA was added to a final concentration of 2% to stop digestion. The resulting peptides were labeled using an on-column TMT labeling protocol^16^. TMT-labeled samples were compiled into a single TMT sample and concentrated. Peptides in the compiled sample were fractionated (6 fractions) using the bRP fractionation. Prior to LC-MS measurement, the peptide fractions were reconstituted in 0.1% TFA, 2% acetonitrile in water. Chromatographic separation was performed on an Easy-Spray Acclaim PepMap column 50 cm long × 75 µm inner diameter (Thermo Fisher Scientific) at 55 °C by applying 90 min acetonitrile gradients in 0.1% aqueous formic acid at a flow rate of 300 nl/min. An UltiMate 3000 nano-LC system was coupled to a Q Exactive HF-X mass spectrometer via an easy-spray source (all Thermo Fisher Scientific). The Q Exactive HF-X was operated in TMT mode with survey scans acquired at a resolution of 60,000 at m/z 200. Up to 18 of the most abundant isotope patterns with charges 2–5 from the survey scan were selected with an isolation window of 0.7 m/z and fragmented by higher-energy collision dissociation (HCD) with normalized collision energies of 32, while the dynamic exclusion was set to 35 s. The maximum ion injection times for the survey and MS/MS scans (acquired with a resolution of 30,000 at m/z 200) were 50 and 150 ms, respectively. The ion target value for MS was set to 3e6 and for MS/MS to 1e5, and the minimum AGC target was set to 1e3.

The data were processed with MaxQuant v. 1.6.17.0^17^, and the peptides were identified from the MS/MS spectra searched against Uniprot *C. elegans* reference proteome (UP000001940) using the built-in Andromeda search engine. Raw files from the LC-MS/MS measurements of 6 tryptic peptide fractions were analyzed together. Reporter ion MS2-based quantification was applied with reporter mass tolerance = 0.003 Da and min. reporter PIF = 0.75. Cysteine carbamidomethylation was set as a fixed modification, and methionine oxidation, glutamine/asparagine deamination, and protein N-terminal acetylation were set as variable modifications. For in silico digests of the reference proteome, cleavages of arginine or lysine followed by any amino acid were allowed (trypsin/P), and up to two missed cleavages were allowed. The FDR was set to 0.01 for peptides, proteins, and sites. A match between runs was enabled. Other parameters were used as pre-set in the software. Unique and razor peptides were used for quantification, enabling protein grouping (razor peptides are the peptides uniquely assigned to protein groups and not to individual proteins). Reporter intensity corrected values for protein groups were loaded into Perseus v. 1.6.10.0^18^. Standard filtering steps were applied to clean up the dataset: reverse (matched to decoy database), only identified by site, and potential contaminant (from a list of commonly occurring contaminants included in MaxQuant) protein groups were removed. Reporter intensity corrected values were log2 transformed, and protein groups with all values were kept. Reporter intensity values were then normalized by median subtraction within TMT channels. Student’s t-test (permutation-based FDR = 0.05, S0 = 0.1) was performed on the dataset to return 0 protein groups, which levels were statistically significantly changed in AHCY-1^Y145C^ vs. AHCY-1 samples. This dataset has been deposited to the ProteomeXchange Consortium^19^ via the PRIDE partner repository^20^ with the dataset identifier PXD041645.

### Microscopy

Young adult worms were immobilized with tetramisole (25 µM) and immediately imaged using a Nikon SMZ25 microscope for GFP fluorescence. For ORO staining, quantification, and data examination, ImageJ (Fiji) was used to process the images, and CTCF values were calculated as described^21^.

### Reporting summary

Further information on research design is available in the Nature Research Reporting Summary linked to this article.

### Supplementary information

Supplementary Information

Supplementary Table 1

Supplementary Table 2

Reporting Summary

## Data Availability

The mass spectrometry proteomics data were deposited to the ProteomeXchange Consortium via the PRIDE partner repository with the dataset identifier PXD041645. The raw data were deposited in Zenodo and are available at 10.5281/zenodo.8392679.
