# Supplementary material for: SAM, SAH and C. elegans longevity: insights from a partial AHCY deficiency model
Source: NPJ Aging. 2023 Dec 5;9(1):27. doi: 10.1038/s41514-023-00125-1 (PMC10698036; doi:10.1038/s41514-023-00125-1)
Supplement: Supplementary file 1 — Supplementary Information [file 41514_2023_125_MOESM1_ESM.pdf]

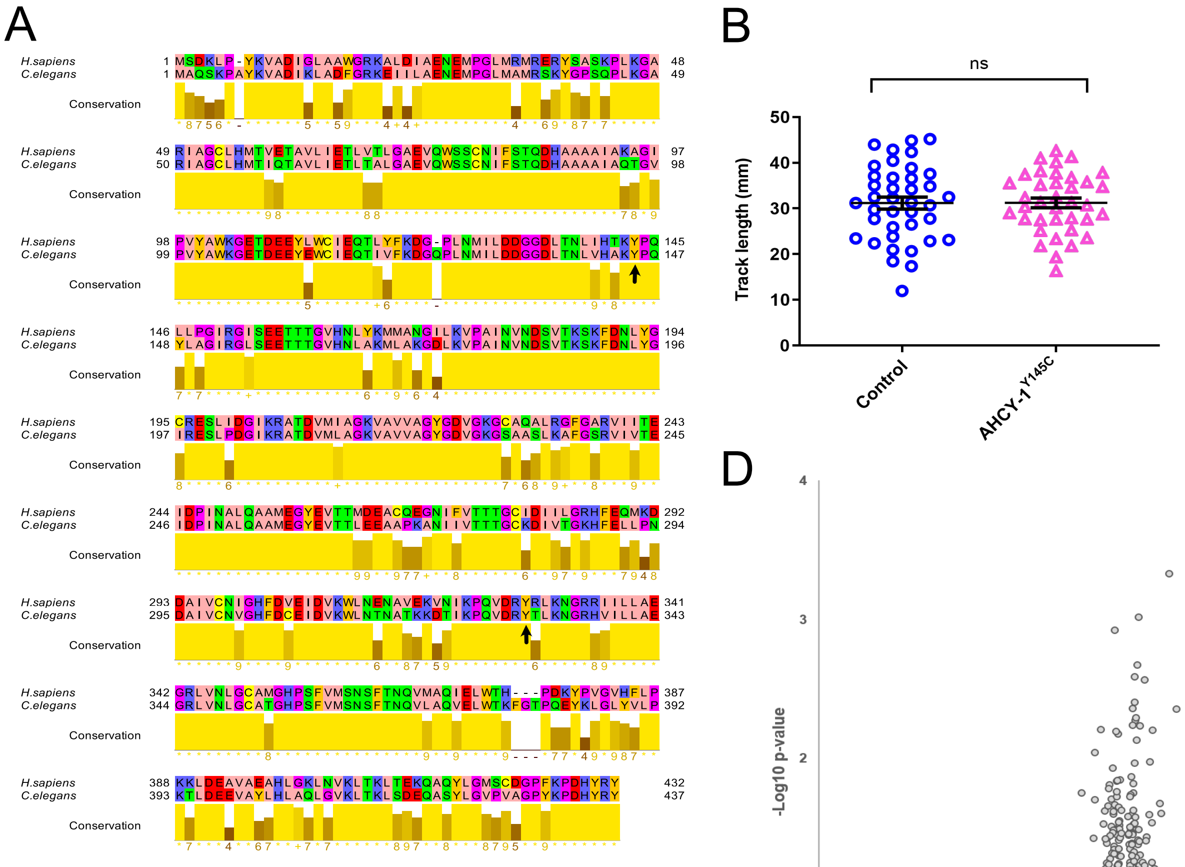

C

	Average number of eggs				Average number of hatched eggs			
	Control	AHCY-1^{Y145C}	Adjusted *p*-value	Significant?	Control	AHCY-1^{Y145C}	Adjusted *p*-value	Significant?
Day 0	8.667	10.44	0.9554	No	8.444	10.33	0.9461	No
Day 1	50.44	53.56	0.6122	No	47.89	53	0.1159	No
Day 2	51.89	54.89	0.6507	No	50.67	51.67	0.998	No
Day 3	40.33	37	0.5345	No	30.33	28.33	0.9301	No
Day 4	15.67	14.67	0.9978	No	12	11	0.998	No
Day 5	2.667	1.556	0.996	No	1.111	1.111	>0.9999	No

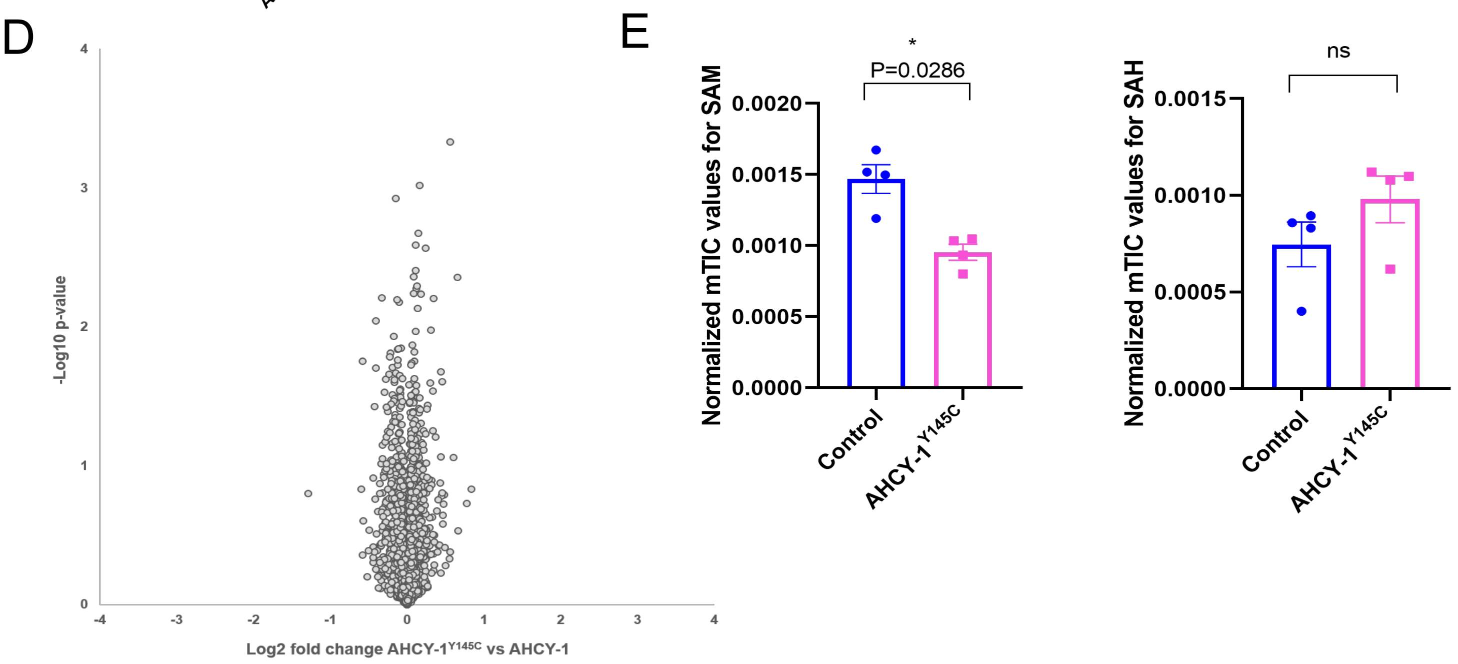

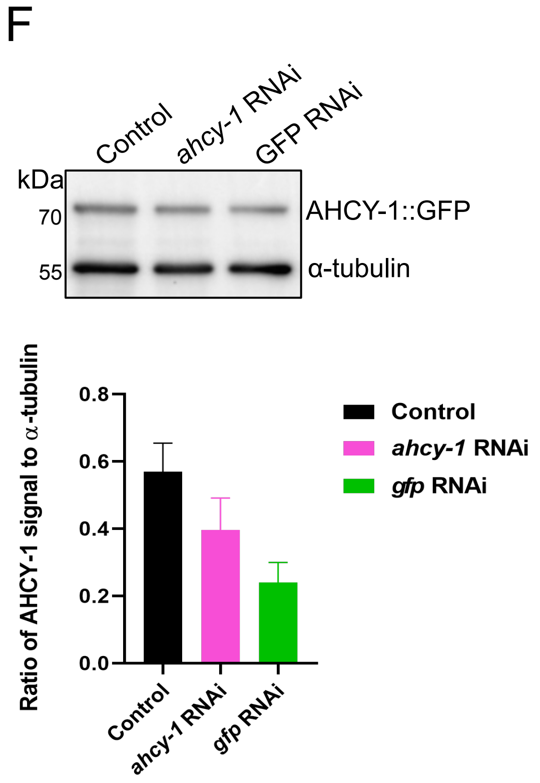

Uncropped immunoblots

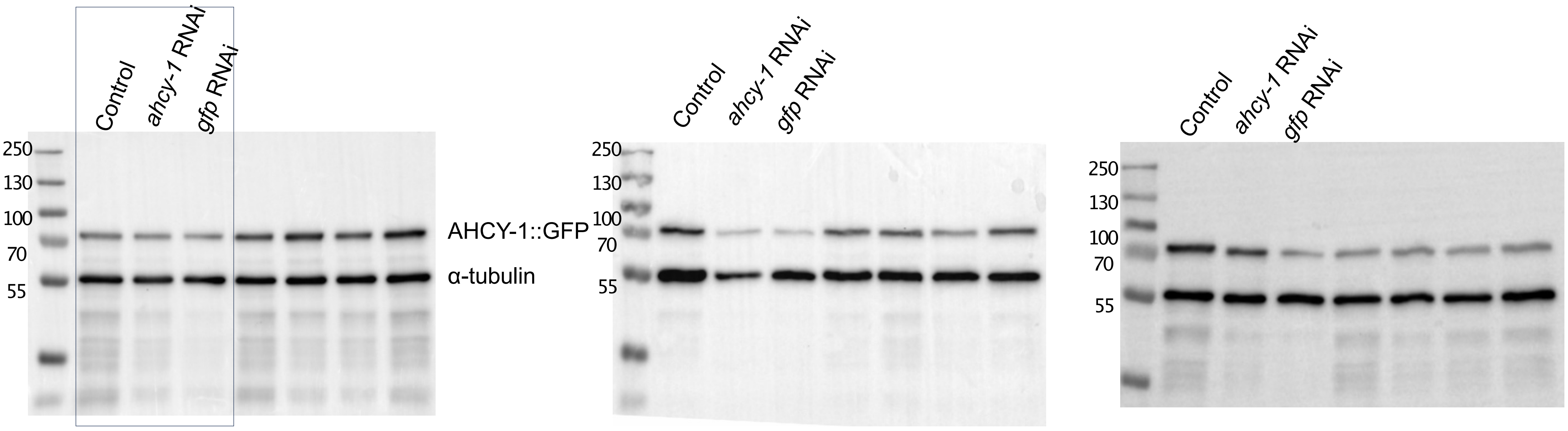

Supplementary Figure 1: Exploring the AHCY-1Y145C mutation and its effects in C. elegans.

A) Sequence alignment of human and C. elegans AHCY obtained from EMBOSS Needle web server, highlighting conservation scores of each residue. Mutant position indicated by an arrow (Y143, Y328 in humans and Y145, Y320 in worms).

B) Comparison of track lengths covered by control and AHCY-1Y145C animals over 2 minutes. Analysis based on 37-39 worms from 3 biological repeats. Statistical significance determined using the Mann-Whitney test.

C) Comparative daily egg-laying and hatching analysis of mutant and wild-type C. elegans over a period of 5 days of adulthood. The table outlines the detailed data gathered daily, documenting the number of eggs laid and subsequently hatched each day for both the wild-type and AHCY-1Y145C mutant strains. Analysis based on 3 biological repeats. Statistical significance determined using the Sidak's multiple comparisons test.

D) Protein levels in AHCY-1Y145C and control whole worm extracts analyzed via TMT shotgun proteomics (from 3 biological repeats). The volcano plot displays the log2 fold change (x-axis) against the t-test-derived −log10 statistical P-value (y-axis) for all protein groups (points on the plot) quantified in the analysis.

E) The LC-MS/MS method was used to measure the levels of SAM and SAH (represented as mTIC-normalized peak intensities) in control (AHCY-1::GFP) and AHCY-1Y145C mutant worm lysates. The mean and SEM were calculated from 4 biological repeats.

F) Quantification and representative immunoblot of AHCY-1::GFP protein levels obtained through western blotting using GFP-specific antibodies. α-tubulin was utilized as a loading control to ensure consistency in the loading quantities across the samples. Alongside the representative immunoblot, we have provided a quantification, showcasing the AHCY-1 signal intensities normalized to the α-tubulin control, based on the aggregate data from three independent experiments. Uncropped immunoblots used for quantification shown below.
