# Supplementary material for: SAM, SAH and C. elegans longevity: insights from a partial AHCY deficiency model
Source: NPJ Aging. 2023 Dec 5;9(1):27. doi: 10.1038/s41514-023-00125-1 (PMC10698036; doi:10.1038/s41514-023-00125-1)
Supplement: Supplementary file 4 — Reporting Summary [file 41514_2023_125_MOESM4_ESM.pdf]

nature portfolio

Corresponding author(s): Wojciech Pokrzywa

Last updated by author(s): 13/09/23

Reporting Summary

Nature Portfolio wishes to improve the reproducibility of the work that we publish. This form provides structure for consistency and transparency in reporting. For further information on Nature Portfolio policies, see our Editorial Policies and the Editorial Policy Checklist.

Statistics

For all statistical analyses, confirm that the following items are present in the figure legend, table legend, main text, or Methods section.

n/a	Confirmed	
☐	☒	The exact sample size (*n*) for each experimental group/condition, given as a discrete number and unit of measurement
☐	☒	A statement on whether measurements were taken from distinct samples or whether the same sample was measured repeatedly
☐	☒	The statistical test(s) used AND whether they are one- or two-sided<br>*Only common tests should be described solely by name; describe more complex techniques in the Methods section.*
☒	☐	A description of all covariates tested
☐	☒	A description of any assumptions or corrections, such as tests of normality and adjustment for multiple comparisons
☐	☒	A full description of the statistical parameters including central tendency (e.g. means) or other basic estimates (e.g. regression coefficient) AND variation (e.g. standard deviation) or associated estimates of uncertainty (e.g. confidence intervals)
☒	☐	For null hypothesis testing, the test statistic (e.g. *F*, *t*, *r*) with confidence intervals, effect sizes, degrees of freedom and *P* value noted<br>*Give P values as exact values whenever suitable.*
☒	☐	For Bayesian analysis, information on the choice of priors and Markov chain Monte Carlo settings
☒	☐	For hierarchical and complex designs, identification of the appropriate level for tests and full reporting of outcomes
☒	☐	Estimates of effect sizes (e.g. Cohen's *d*, Pearson's *r*), indicating how they were calculated

Our web collection on statistics for biologists contains articles on many of the points above.

Software and code

Policy information about availability of computer code

Data collection	Nikon SMZ25 microscope, Q Exactive HF-X mass spectrometer via an easy-spray source (all Thermo Fisher Scientific), Vanquish UHPLC system coupled to Orbitrap Exploris 240 high-resolution mass spectrometer (Thermo Scientific), WormLab system (MBF Bioscience).
Data analysis	Data were analysed using GraphPad Prism 9 (GraphPad Software, Inc.), Office 365 (Microsoft), MaxQuant v. 1.6.17, Bio-Rad ImageLab, WormLab software (MBF Bioscience), and ImageJ (Wayne Rasband). Data were visualised using GraphPad Prism 9 (GraphPad Software, Inc.), and Affinity Designer.

For manuscripts utilizing custom algorithms or software that are central to the research but not yet described in published literature, software must be made available to editors and reviewers. We strongly encourage code deposition in a community repository (e.g. GitHub). See the Nature Portfolio guidelines for submitting code & software for further information.

Data

Policy information about availability of data

All manuscripts must include a data availability statement. This statement should provide the following information, where applicable:

- Accession codes, unique identifiers, or web links for publicly available datasets
- A description of any restrictions on data availability
- For clinical datasets or third party data, please ensure that the statement adheres to our policy

The mass spectrometry proteomics data were deposited to the ProteomeXchange Consortium via the PRIDE partner repository with the dataset identifier

PXD041645. The authors declare that the main data supporting the findings of this study are available within the article and its supplementary files. All the other data are also available from the corresponding authors upon request.

Research involving human participants, their data, or biological material

Policy information about studies with human participants or human data. See also policy information about sex, gender (identity/presentation), and sexual orientation and race, ethnicity and racism.

Reporting on sex and gender	N.A.
Reporting on race, ethnicity, or other socially relevant groupings	N.A.
Population characteristics	N.A.
Recruitment	N.A.
Ethics oversight	N.A.

Note that full information on the approval of the study protocol must also be provided in the manuscript.

Field-specific reporting

Please select the one below that is the best fit for your research. If you are not sure, read the appropriate sections before making your selection.

☒ Life sciences ☐ Behavioural & social sciences ☐ Ecological, evolutionary & environmental sciences

For a reference copy of the document with all sections, see nature.com/documents/nr-reporting-summary-flat.pdf

Life sciences study design

All studies must disclose on these points even when the disclosure is negative.

Sample size	No statistical methods were used to predetermine sample sizes. Sample sizes are similar to those used in the field (as previously reported: Turek et al. EMBO Reports 2020) and were done according to standard C. elegans approaches. Exact sample sizes are indicated in the corresponding figure legends and Supplementary Table 2.
Data exclusions	No data were excluded from the analysis.
Replication	Each experiment was repeated at least three times in independent experiments in multiple animals. Experimental findings were reproduced reliably.
Randomization	Worms were randomly allocated to the experimental groups for all the data sets.
Blinding	Investigators were blinded to group allocation during data collection and analysis for most of the experiments performed within this study. A list of blinded experiments is presented in the Methods section. Blinding was not performed only for these experiments which were performed by the same investigator throughout the whole procedure and because of the unambiguous nature of measurements and systematic analysis used in these experiments. However, the different conditions were assessed in random order.

Reporting for specific materials, systems and methods

We require information from authors about some types of materials, experimental systems and methods used in many studies. Here, indicate whether each material, system or method listed is relevant to your study. If you are not sure if a list item applies to your research, read the appropriate section before selecting a response.

Materials & experimental systems

n/a	Involved in the study	
☐	☒	Antibodies
☒	☐	Eukaryotic cell lines
☒	☐	Palaeontology and archaeology
☐	☒	Animals and other organisms
☒	☐	Clinical data
☒	☐	Dual use research of concern
☒	☐	Plants

Methods

n/a	Involved in the study	
☒	☐	ChIP-seq
☒	☐	Flow cytometry
☒	☐	MRI-based neuroimaging

Antibodies

Antibodies used	anti-α-tubulin antibody (Sigma-Aldrich, T6074, 1:10 000 dilution) and anti-Green Fluorescent Protein (Bio-Rad, AHP975, 1:2000 dilution).
Validation	The antibodies have already been validated many times, which can be confirmed on the manufacturers' website. The validation in this manuscript is shown in Figure S1F.

Animals and other research organisms

Policy information about studies involving animals; ARRIVE guidelines recommended for reporting animal research, and Sex and Gender in Research

Laboratory animals	In this study, we used the nematode Caenorhabditis elegans.<br>C. elegans strains: N2 (wild type), PHX646 (ahcy-1(syb646[ahcy-1::GFP]I). The strain generated in this study, PHX784 (syb784 syb646 [ahcy-1Y156C::GFP]I), was produced by SunyBiotech using CRISPR services (http://www.sunybiotech.com). PHX646 and PHX784 were outcrossed 2X to N2 to generate WOP122 and WOP159 strains, respectively. All strains are listed in the Methods section where their genotype is also described.
Wild animals	The study did not involve wild animals.
Reporting on sex	The study was performed on C.elegans hermaphrodites.
Field-collected samples	The study did not involve samples collected from the field
Ethics oversight	Studies on the namatode Caenorhabditis elegans require no ethical approval.

Note that full information on the approval of the study protocol must also be provided in the manuscript.
